# LncRNAs regulate ferroptosis to affect diabetes and its complications

**DOI:** 10.3389/fphys.2022.993904

**Published:** 2022-09-26

**Authors:** Qianqian Chen, Hao Ji, Yue Lin, Zheyan Chen, Yinai Liu, Libo Jin, Renyi Peng

**Affiliations:** ^1^ Institute of Life Sciences and Biomedicine Collaborative Innovation Center of Zhejiang province, College of Life and Environmental Science, Wenzhou University, Wenzhou, China; ^2^ Department of Emergency, Wenzhou People’s Hospital, The Third Affiliated Hospital of Shanghai University and Wenzhou Third Clinical Institute Affiliated to Wenzhou Medical University, Wenzhou, China; ^3^ Department of Plastic Surgery, Wenzhou People’s Hospital, The Third Affiliated Hospital of Shanghai University and Wenzhou Third Clinical Institute Affiliated to Wenzhou Medical University, Wenzhou, China

**Keywords:** lncRNAs, ferroptosis, diabetes, Nrf2, NF-κB

## Abstract

Worldwide, the rapid increase in the incidence of diabetes and its complications poses a serious threat to human health. Ferroptosis, which is a new nonapoptotic form of cell death, has been proven to be closely related to the occurrence and development of diabetes and its complications. In recent years, lncRNAs have been confirmed to be involved in the occurrence and development of diabetes and play an important role in regulating ferroptosis. An increasing number of studies have shown that lncRNAs can affect the occurrence and development of diabetes and its complications by regulating ferroptosis. Therefore, lncRNAs have great potential as therapeutic targets for regulating ferroptosis-mediated diabetes and its complications. This paper reviewed the potential impact and regulatory mechanism of ferroptosis on diabetes and its complications, focusing on the effects of lncRNAs on the occurrence and development of ferroptosis-mediated diabetes and its complications and the regulation of ferroptosis-inducing reactive oxygen species, the key ferroptosis regulator Nrf2 and the NF-κB signaling pathway to provide new therapeutic strategies for the development of lncRNA-regulated ferroptosis-targeted drugs to treat diabetes.

## Introduction

Diabetes is characterized by many complications, including prolonged illness, slow onset and long disease course, and can cause complications in the kidney, nerves, blood vessels and other organs, which affects the quality of life of patients and has become a serious public health problem. For patients with diabetes, long-term hyperglycemia can cause a variety of acute and chronic complications, such as cardiovascular disease, stroke, diabetic foot ulcer, diabetic ketoacidosis and other diseases. At present, diabetes is controlled through drugs, diet control and insulin injections, but there are still shortcomings, such as insulin resistance, the short duration of drug effects and unstable blood glucose maintenance. Due to the complex etiology and unclear pathogenesis of diabetes, it is difficult to develop effective treatment drugs and strategies (Bloomgarden, 2004; Cole and Florez, 2020; Ising et al., 2021). Therefore, further in-depth study of the pathogenesis of diabetes is necessary. With the deepening of research, new pathological mechanisms have been examined; ferroptosis is involved in the occurrence and development of diabetes, and long noncoding RNAs (lncRNAs) are also involved in regulating this process.

Ferroptosis, which is a newly defined programmed cell death that is different from apoptosis and autophagy, is characterized by intracellular iron overload and the accumulation of reactive oxygen species (ROS), leading to lipid peroxidation. Ferroptosis involves abnormal intracellular lipid metabolism under the catalysis of iron ions (Latunde-Dada, 2017; Jiang et al., 2021). When the antioxidant capacity of cells weakens and excessive lipid reactive oxygen species accumulate, intracellular redox imbalance and cell death occur, which is also the essential difference between ferroptosis and necrosis, apoptosis and autophagy. Moreover, ferroptosis has been confirmed to be involved in regulating the development of a variety of diseases, including cancer, neurodegenerative diseases, cardiomyopathy, stroke, brain injury, ischemia–reperfusion injury, atherosclerosis and acute renal failure (Li J. et al., 2020; Qiu et al., 2020). An increasing number of studies have proven that ferroptosis is closely related to the occurrence and development of diabetes and its complications. Therefore, in-depth study of the mechanism of ferroptosis-mediated diabetes and its complications will shed new light on the prevention and treatment of diabetes. Ferroptosis plays an important regulatory role in diabetic nephropathy (DN), diabetic cardiomyopathy (DCM), diabetic osteoporosis (DOP), diabetic retinopathy (DR), and diabetic peripheral neuropathy (DPN).

The main mechanisms of ferroptosis can be divided into the iron metabolism pathway, the glutathione (GSH)/glutathione peroxidase 4 (GPX4) pathway and the lipid metabolism pathway, which are affected by cystine/glutamate anti-porter system xc- (system xc-), GSH, the GPX4 signaling pathway and polyunsaturated fatty acid (PUFA) levels. Fe^2+^ accumulates in the cell, the Fenton reaction leads to the production of a large amount of ROS, and ROS-induced lipid peroxidation results in the production of malonaldehyde (MDA) and 4-HNE, which are toxic to cells and ultimately induce ferroptosis. In addition, regulating intracellular iron levels can change the sensitivity of cells to ferroptosis by increasing transferrin and transferrin receptor-1 to increase intracellular iron levels and promote ferroptosis (Park and Chung, 2019; He et al., 2020; Sha et al., 2021). Moreover, GPX4 is an inhibitory protein in lipid peroxidation that reduces lipid peroxides to lipid alcohols, thus preventing the accumulation of ROS and inhibiting ferroptosis. The activity of GPX4 depends on the exchange of extracellular cystine and intracellular glutamate and is mediated by system xc-, an amino acid antiporter that is widely distributed in the bilayer of phospholipids. Cystine is reduced from l-cysteine and is the substrate for GSH synthesis, and GSH is a necessary cofactor for GPX4. Therefore, the accumulation of iron and lipid peroxides is one of the main causes of ferroptosis in cells (Ingold et al., 2018; Forcina and Dixon, 2019; Ursini and Maiorino, 2020). However, free PUFAs are prone to lipid peroxidation and are substrates of synthetic lipid signal transduction. PUFA abundance and localization determine the degree of lipid peroxidation in cells, which determines the effect of ferroptosis. The downregulation of GPX4 expression, the clearance of lipid reactive oxygen species and an increase in mRNA levels are all key characteristics of ferroptosis and related indicators of diabetes and its complications (Han et al., 2020; Ajoolabady et al., 2021; Qi et al., 2021).

In recent years, as a research hotspot, lncRNAs have attracted wide attention because of their effects on a variety of molecular regulatory pathways. Long noncoding RNAs (lncRNAs) are defined as RNAs whose transcripts are more than 200 nucleotides in length and are not translated into proteins. In addition, lncRNAs can encode small peptides that fine-tune general biological processes in a tissue-specific manner. LncRNAs have been shown to regulate cellular processes, such as chromosome and genome modifications, transcriptional activation and interference, and nuclear trafficking, thus prompting more researchers to explore how lncRNAs affect human biology (Chen et al., 2021d). LncRNAs are widely involved in all aspects of cellular functions, regulating related protein coding genes at different levels in a variety of ways and affecting the expression of target genes. LncRNAs are involved in the occurrence and development of various diseases, such as tumors and cancers, and play an important regulatory role in the occurrence and development of diabetes and its complications. In addition, an increasing number of studies have shown the involvement of lncRNAs in regulating diabetes. Studies have shown that the occurrence and development of diabetes are closely related to the abnormal expression of many kinds of lncRNAs, but the specific regulatory mechanism is still unclear. Therefore, the regulatory relationship between lncRNAs and diabetes and its complications has become another hot spot in the field of diabetic pathology. Due to the occult nature of lncRNA expression, the tissue specificity of expression cannot be timely and accurately determined by routine diabetes examination, but lncRNAs are highly stable in eukaryotic cells and the circulatory system, so they can be used as early molecular markers of diabetes (Suwal et al., 2019; Thomas et al., 2019; Feng Y. et al., 2019; He et al., 2021).

Because the regulation of lncRNAs and ferroptosis affect the occurrence and development of diabetes, whether lncRNAs regulate ferroptosis-mediated diabetes still needs to be further investigated. This review summarizes the effects of lncRNA regulation and the ferroptosis pathway on the development of diabetes and the role of these factors in the treatment of diabetes. We focused on the pathogenesis of ferroptosis pathway-mediated diabetes and its complications, which are regulated by lncRNAs, to provide valuable reference information for the treatment of clinical diabetes and the creation of new drugs.

## Ferroptosis and lncRNA affect the development of diabetes and its complications

### The role of ferroptosis in the development of diabetes and its complications

The occurrence of ferroptosis involves the expression and regulation of many genes, and it signaling pathway is complex. To date, accumulating evidence shows that ferroptosis is one of the causes of diabetes and its complications, and controlling the factors that affect ferroptosis may be an effective strategy for the early diagnosis and treatment of diabetes and its complications. Therefore, further research on ferroptosis is needed to identify therapeutic targets for diabetes and its complications (Yang and Yang, 2022).

Persistent inflammation induced by hyperglycemia is one of the characteristics of diabetic patients, and ferroptosis is closely related to diabetic inflammation. When an immunodeficient mouse model was established and the pancreatic islet was treated with ferroptosis inhibitors, pancreatic islet transplantation and function were not affected, suggesting that ferroptosis affected pancreatic islet function (Karam et al., 2017; Badgley et al., 2020; Kremer et al., 2021). In addition, in response to arsenic exposure, the mitochondrial membrane potential and cytochrome C levels in islet cells decreased, while ROS levels increased, suggesting that arsenic can induce ferroptosis in the pancreatic islet (Wei et al., 2020; Yao et al., 2021; Li et al., 2022a).

In DN studies, ROS accumulation and iron overload are important determinants of DN. The expression level of ACSL4 was increased and GPX4 was decreased in DN mice, and the levels of lipid peroxidation products and iron were increased, suggesting that ferroptosis was involved in the development of DN. However, the ACSL4 inhibitor rosiglitazone could inhibit the production of proinflammatory cytokines and the development of DN by reducing the levels of lipid peroxidation products, MDA and iron, leading to the blockade of ferroptosis in renal tubular cells (Ferrè et al., 2016; Kim et al., 2021; Huang et al., 2022; Wu et al., 2022).

In patients with DCM, ferroptosis and apoptosis may occur in cardiomyocytes because the imbalance in the antioxidant system usually leads to excessive ROS production. Studies have shown that ferroptosis-1 (Fer-1) can alleviate myocardial injury by inhibiting ferroptosis during myocardial ischemia and reperfusion in diabetic rats (Feng et al., 2017; Yu et al., 2018; Wang X. et al., 2022). Das and others have shown that resveratrol can improve cardiomyocyte fibrosis caused by iron overload, thus alleviating DCM(Li et al., 2022b).

In addition, DOP is associated with ferroptosis. Iron overload promotes osteoclast differentiation and bone resorption through ROS production (Yang et al., 2022b). When Fer-1 was used to inhibit osteoblast-related proteins in Mc3t3-e1 cells under high glucose conditions, the protein expression levels of osseous and osteocalcin were downregulated, suggesting that ferroptosis affects osteogenesis and the development of DOP in response to high glucose (Feng et al., 2022).

DR is a chronic complication of diabetes mellitus, and its pathogenesis is closely related to ferroptosis. The retina is vulnerable to oxygen free radical damage, and oxidative damage caused by free radicals produced by Fe^2+^ during the oxidation process is considered to be key to DR pathogenesis. Increased iron levels in the eyes of diabetic patients lead to oxidative damage to photoreceptors, and reduced glutathione and other antioxidants will be consumed in large quantities, resulting in increased ferroptosis (Chen et al., 2021a; Tang et al., 2021).

**FIGURE 1:**
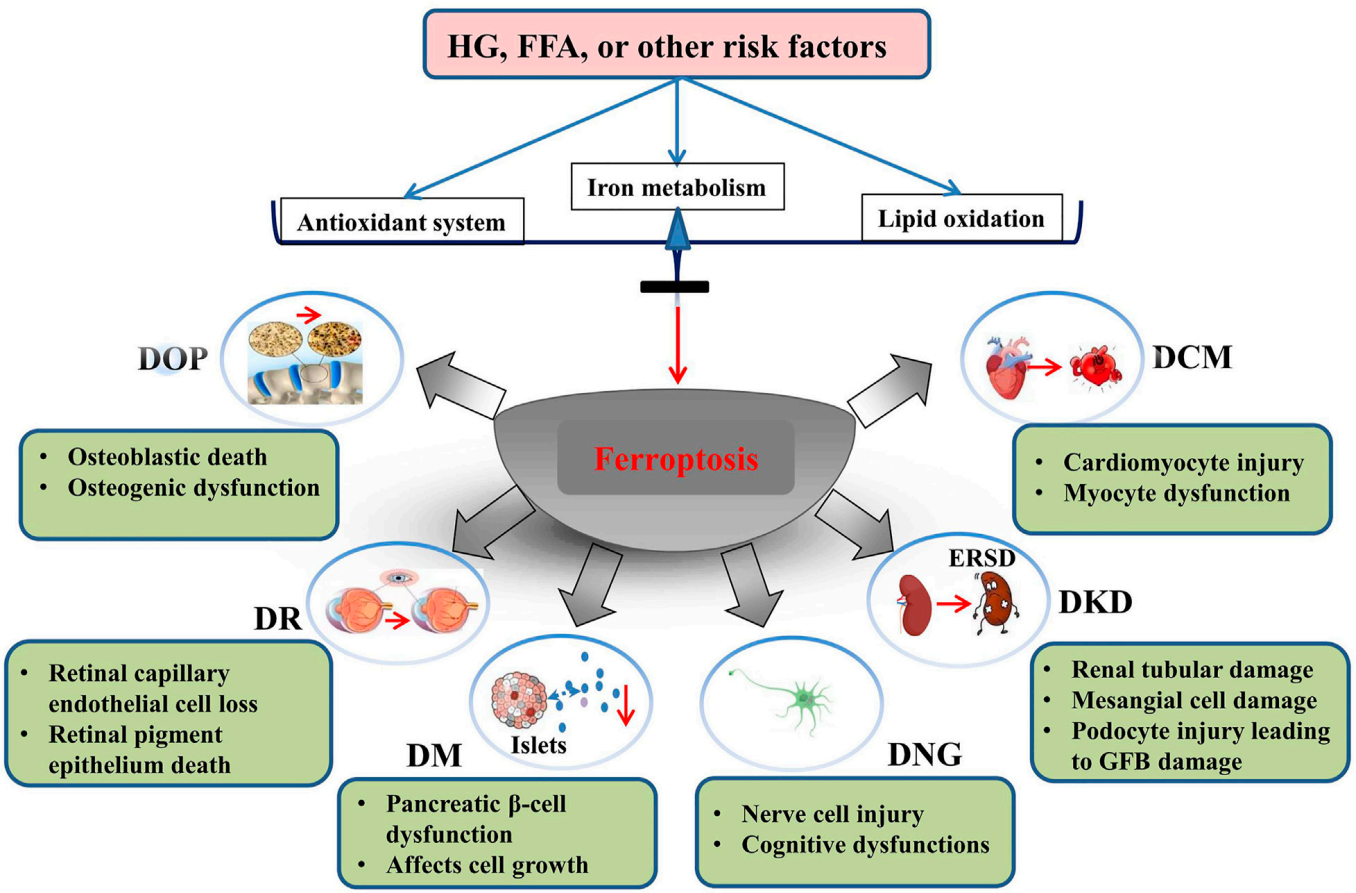
Role of ferroptosis in the development of diabetes mellitus and its complications. HG, high glucose; FFA, free fatty acids; DOP, diabetic osteoporosis; DR, diabetic retinopathy; DM, diabetes mellitus; DNG diabetic neuropathy; DKD, diabetic kidney disease; DCM, diabetic cardiomyopathy. Reproduced with permission from (Yang and Yang, 2022). Copyright 2022, Frontiers in Endocrinology.

During the development of DNG, iron is redistributed in specific areas of the central and peripheral nervous systems. Paeschke and others have shown that the levels of blood glucose and glycosylated hemoglobin in the high-iron group are reduced, while the concentration of insulin was increased (Morsi et al., 2018; Paeschke et al., 2019). Dietary supplementation with nonheme iron can partially improve neurological dysfunction in diabetic mice. The mechanism may be that supplementation with nonheme iron reduces the activity of proinflammatory cytokines in the peripheral nervous system (Baum et al., 2016, 2021; He et al., 2022). Therefore, a large number of studies have shown that ferroptosis plays an important role in the pathogenesis of diabetes and its complications (Figure 1).

### The role of lncRNAs in the development of diabetes and its complications

As scientists studied genes further, they found that only 2% of the 3 billion base pairs in the human genome can encode proteins. The remaining 98% lack the ability to encode proteins and were called noncoding RNAs (ncRNAs). NcRNAs can be divided into microRNAs (miRNAs) and lncRNAs according to fragment size (Xu et al., 2010). LncRNAs regulate related protein-coding genes in various ways at different levels and can complement DNA bases to form stable triple-helix complexes and participate in cell proliferation, differentiation and apoptosis (Bell et al., 2014; Divoux et al., 2014; Bridges et al., 2021). Evidence shows that abnormal lncRNA expression is closely related to the occurrence and development of diabetes and its complications and can be a potential therapeutic target.

The impairment or loss of islet cell function is the direct cause of diabetes, and lncRNAs play an important role in regulating insulin-secreting *β* islet cells (Table 1) (Marrero et al., 2005; Hammes et al., 2011; Fadista et al., 2014; Hu et al., 2014; Li et al., 2014; You et al., 2015). During insulin secretion and synthesis, the transcription factor Glut2, which controls transport, and the transcription factors Pdx1, MafA and NeuroD, which control synthesis, are regulated by lncRNA TUG1, and interference with lncRNA TUG1 leads to diabetes. When the expression level of lncRNA TUG1 in the pancreas is decreased, the insulin secretion and synthesis of islet *β* cells is decreased, and the regulation of blood glucose is weakened (Morán et al., 2012; Yin et al., 2015; Zhang et al., 2021). In addition, lncRNAs were shown to have therapeutic effects on type 2 diabetes mellitus (T2DM) patients during liver glycogen synthesis. During hyperglycemia, the expression of lncRNA-N021972 in the livers of T2DM rats was increased. However, lncRNA-N021972 siRNA could increase the level of phosphorylated protein kinase B, upregulate the expression of hepatic glucokinase, and increase the synthesis of liver glycogen, thereby reducing hyperglycemia levels in T2DM rats (Taheri et al., 2020).

**TABLE 1:** LncRNAs are involved in the occurrence and development of diabetes. Adapted with permission from (Feng et al., 2017). Copyright 2016, Biochemistry and Cell Biology.

LncRNAs	Chromosome (species)	Type	Target gene	Function	References
HI-LNC25	Chr20 (human)	Intergenic	GLIS3	HI-LNC25 positively regulates GLIS3 mRNA, which contains risk variants for T1D and T2D	Morán et al. (2012)
LOC283177	Chr11 (human)	Intergenic	N.A.	XLOC-019089 is strongly coexpressed with SYT11, MADD, and PAX6 to regulate the production and secretion of insulin	Fadista et al. (2014)
SRA	Chr18 (mouse)	N.A.	PPARγ, p21Cip1, and p27Kip1	SRA promotes adipogenesis by transcriptionally coactivating PPARγ, promoting S-phase entry and regulating adipocyte cycle-related genes	Liu et al. (2014), Xu et al. (2010)
SRA	Chr18 (mouse)	N.A.	pAkt, GLUT3, and SLC2A3	SRA increases insulin-induced glucose uptake *in vitro*; SRA gene knockout improves glucose tolerance in mice fed an HFD	Foulds et al. (2010), Liu et al. (2014), Xu et al. (2010)
SRA	Chr18 (mouse)	N.A.	pAkt, TNFα	SRA increases insulin-induced glucose uptake *in vitro*; SRA gene knockout improves glucose tolerance in mice fed an HFD	Liu et al. (2014), Xu et al. (2010)
HOTAIR	Chr18 (mouse)	N.A.	PPARγ, LPL	HOTAIR promotes adipogenesis by regulating key adipogenic genes PPARγ and LPL	Divoux et al. (2014)
PU.1AS	Chr2 (mouse) & Chr2 (pig)	Antisense	PU.1	PU.1 AS promotes adipogenesis by preventing PU.1 mRNA translation by forming the PU.1 mRNA-PU.1 AS lncRNA duplex	Pang et al. (2013); Wei et al. (2020)
Binc1	Chr14 (mouse)	Intergenic	EBF2	Blnc1 forms a feedforward regulatory loop with EBF2 that directs adipogenesis toward thermogenesis	Zhao et al. (2014)
CRNDE	Chr16 (human)	Intergenic	N.A.	CRNDE is a downstream target of the PI3K-Akt-mTOR and Raf-MAPK pathways and regulates genes involved in central metabolism	Ellis et al. (2014)
UCA1	Chr19 (human)	N.A.	HK2	UCA1 promotes glucose consumption and lactate production through the mTOR-STAT3-miR143-HK2 pathway in cancer cells	Li et al. (2014)
LincRNA-DYNLRB2-2	Chr16 (human)	Intergenic	GPR119	LincRNA-DYNLRB2-2 modulates the GPR119-GLP-1R-ABCA1-dependent signal transduction pathway to regulate cholesterol homeostasis	Hu et al. (2014)
lncLSTR	Chr1 (mouse)	N.A.	ApoC2	IncLSTR modulates the FXR-apoC2PLP pathway by regulating TDP-43-Cyp8b1 to maintain systemic lipid homeostasis	Li et al. (2015)
TUG1	Chr1 (mouse)	N.A.	N.A.	Downregulation of lncRNA TUG1 expression increases apoptosis and reduces insulin secretion in islet *β*-cells *in vitro* and *in vivo*	Yin et al. (2015)
Meg3	Chr12 (mouse)	N.A.	Pdx-1, MafA	Meg3 regulates *β*-cell identity and function by affecting insulin production and apoptosis	You et al. (2016)

Studies have shown that lncRNAs have therapeutic effects on diabetic complications. In DN, lncRNA TUG1 alleviates the progression of DN by targeting miR-377 and thereby inhibiting ECM accumulation. During the repair of diabetic inflammatory wounds, miR-17, miR-18a, miR-19a, miR-19b and miR-20a expression levels are upregulated (Pang et al., 2013; Li et al., 2015; Lei et al., 2018). MiR-19a/b and miR-20a reduce the production of inflammatory chemokines and cytokines by targeting SHCBP1 and SEMA7A, respectively, thus promoting diabetic wound healing. LncRNA MALAT1 was significantly upregulated in mice with diabetic cardiomyopathy and in cardiomyocytes induced by high glucose, and knocking down lncRNA MALAT1 expression could significantly improve apoptosis induced by high glucose and inhibit mir-181A-5p expression (Michalik et al., 2014; Leung and Natarajan, 2018; Zhang et al., 2019b; Li et al., 2021a; Tanwar et al., 2021). In addition, other lncRNAs play certain roles in the occurrence and development of diabetes and related diseases and can be used as therapeutic targets for diabetic complications (Table 2) (Foulds et al., 2010; Ellis et al., 2014; Liu et al., 2014; Li et al., 2015).

**TABLE 2:** LncRNAs are involved in the occurrence and development of diabetic complications. Adapted with permission from (Feng et al., 2017). Copyright 2016, Biochemistry and Cell Biology.

LncRNAs	Chromosome (species)	Type	Target gene	Function	References
MALAT1	Chr11 (mouse)	Intergenic	CPNL1	MALAT1 is a potential biomarker of DR and is significantly upregulated in the RF/6 A cell model of hyperglycemia, as well as in the aqueous humor and fibrovascular membranes of diabetic patients	Lange et al. (2012); Yamamoto et al., 1003
MALAT1	Chr11 (mouse)	N.A.	SAA3	MALAT1 regulates glucose-induced inflammatory changes and oxidative stress via SAA3 in the kidney in diabetic mice	Hammes et al. (2011)
PVT1	Chr8 (Human)	N.A.	FN1, COL4A1, TGFB1, and PAI-1	PVT1 is associated with ESRD in T1D and T2D, probably by mediating extracellular matrix accumulation in the kidney	Bell et al. (2014); Marrero et al. (2005); Michalik et al. (2014); Wu et al. (2022)

## LncRNAs regulate the development of ferroptosis-mediated diabetes and its complications

### LncRNAs affect the development of diabetes and its complications by regulating ROS-induced ferroptosis

Intracellular ROS play a key role in ferroptosis, and lncRNAs can affect the occurrence of ferroptosis by regulating the levels of cellular ROS, thus affecting the development of diabetes and its complications. Recent studies have shown that the ferroptosis inducer erastin causes cancer cells to die due to the accumulation of lipid oxides. The negative regulatory factors MDM2 and MDMX of the tumor suppressor gene p53 can promote ferroptosis through lipid metabolism mediated by PPARα. Therefore, the accumulation of intracellular lipid peroxides is one of the main factors that triggers ferroptosis (Su et al., 2019; Yang et al., 2022a). Increasing evidence shows that islet *β*-cell death induced by high glucose is caused by excessive ROS production, which is caused by intracellular iron deposition and leads to oxidative stress. ROS production is increased and antioxidants are decreased in glomerular mesangial cells and renal tubular epithelial cells induced by high glucose, resulting in ferroptosis (Miess et al., 2018; Chen et al., 2021c; Li et al., 2022c). Therefore, the accumulation of ROS is not only the main cause of ferroptosis but also one of the causes of islet *β*-cell death and leads to ferroptosis, which exacerbates the damage associated with diabetes and its complications.

Some lncRNAs can regulate ROS levels, reduce the incidence of ferroptosis and affect the development of diabetes. In DKD, antisense mitochondrial noncoding RNA-2 (ASncmtRNA-2) was upregulated, and lncRNA ASncmtRNA-2 was significantly downregulated in response to the ROS inhibitor NG-nitro-l-arginine methyl ester (L-NAME). In addition, in high glucose-cultured MCs, the expression level of lncRNA ASncmtRNA-2 was upregulated, and after MCs were cultured L-NAME, the upregulation of lncRNA ASncmtRNA-2 was decreased, indicating that lncRNA ASncmtRNA-2 may be the substrate of ROS and plays an ROS-mediated role in DKD. Through further study, it was found that the mRNA expression level of lncRNA ASncmtRNA-2 was positively correlated with the mRNA expression level of transforming growth factor-β1 (TGFβ1). When lncRNA ASncmtRNA-2 was knocked down, the mRNA levels of TGFβ1 and FN were significantly decreased. Thus, the expression of lncRNA ASncmtRNA-2 can prevent the accumulation of ROS and improve fibrosis in DN (Borgna et al., 2017; Gao et al., 2017).

The latest research confirms that lncRNA NEAT1 plays a role in DN. In response to high glucose conditions, the level of lncRNA NEAT1 in human kidney-2 (HK-2) cells was upregulated, while the level of miR-150-5p was downregulated. Knockout of lncRNA NEAT1 inhibited the excessive production of ROS by HK-2 cells and exacerbated the occurrence of ferroptosis, which led to renal function damage and further accelerated the course of DN (Ruan et al., 2019; Wang et al., 2019; Liao et al., 2020). Studies have confirmed that the level of lncRNA TUG1 in the renal tissue and cells of DKD mice was decreased. LncRNA TUG1 can target and negatively regulate the expression of miR-34a-5p, reduce the levels of ROS and MDA and increase the levels of SOD, thus regulating the level of oxidative stress, promoting the occurrence of ferroptosis, and exacerbating the deterioration of renal pathological structure. High expression of miR-34a-5p can reverse the effect of lncRNA TUG1 on the levels of ROS, MDA and SOD and alleviate the occurrence of DKD (Long et al., 2016; Li et al., 2020d, Li et al., 2021c). It has been reported that the upregulation of lncRNA Blnc1 expression can reduce inflammation and oxidative stress injury, thus improving renal fibrosis and alleviating DN. In addition, increased expression of lncRNA Blnc1 was observed in the STZ-induced DKD rat model and HK2 cells treated with high glucose *in vitro*. Upregulation of lncRNA Blnc1 expression could significantly decrease the levels of PTEN, cellulose, collagen I, collagen IV, inflammatory factors (TNF-α, IL-6, IL-1β) and ROS in HK-2 cells, inhibit the occurrence of ferroptosis, and alleviate the occurrence of diabetes and its complications (Zhao et al., 2014; Feng X. et al., 2019; Xie et al., 2021).

Therefore, the accumulation of large amounts of ROS and iron overload are important determinants of diabetes, and the accumulation of ROS is regulated by lncRNAs significantly and affects the development of diabetes, suggesting that targeted intervention of ferroptosis by regulating lncRNAs is a therapeutic strategy for treating diabetes and its complications.

### LncRNA affects the development of diabetes and its complications by regulating the key ferroptosis regulator Nrf2

The nuclear factor erythroid 2-related factor 2 (Nrf2) signaling pathway is a key pathway associated with cellular antioxidant activity, is thought to play an important role in T2DM and has become a key regulator of lipid peroxidation and ferroptosis. In addition, Nrf2 is closely related to ferroptosis in the development of diabetes. Nrf2 upregulation can slow the progression of diabetes by inhibiting ferroptosis. In a high glucose environment, specific gene knockout of Nrf2 increased the sensitivity of cells to ferroptosis (Junduo et al., 2020; Zang et al., 2020). In the high glucose environment, specific gene knockout of Nrf2 increased the sensitivity of cells to ferroptosis. Immunofluorescence analysis showed that the fluorescence intensity of renal tubules in diabetic mice was significantly weaker than that in normal mice, indicating that the protein expression of Nrf2 in diabetic patients was reduced. Li et al. found that the protein and mRNA expression levels of Nrf2 in the renal tubules of diabetic mice in the DM group were significantly lower than those in the NC group, as determined by immunofluorescence analysis, western blotting and qRT–PCR analysis (Dodson et al., 2019; Wang et al., 2021; Zhao et al., 2021). When Nrf2 was knocked down in cells, the protein expression levels of GPX4, SLC7A11 and FTH-1 were significantly reduced and TFR-1 was increased. These studies showed that low expression of Nrf2 enhanced sensitivity to ferroptosis. Fenofibrate treatment can inhibit the occurrence of cell iron death by upregulating Nrf2 expression, thus delaying the development of DN. In a mouse DN model, treatment with Fer-1 reduced renal pathological damage, and Nrf2 was observed in the DN model (Li et al., 2021d).

Nrf2 is the key regulator that maintains the oxidative stability of cells, and iron overload-related genes and lipid peroxidation genes are also target genes of Nrf2. Nrf2 accumulation and nuclear translocation can be promoted by oxidative stress or other stimuli. When Nrf2 enters the nucleus, it binds to a small Maf protein to activate the antioxidant/electrophilic response element in the regulatory region of antioxidant enzyme genes to eliminate ROS and maintain cell redox homeostasis. Heme oxygenase-1 (HO-1) is a key antioxidant enzyme that is regulated by Nrf2 (Fan et al., 2017; Anandhan et al., 2020; Ma et al., 2020; Li et al., 2021b). Nrf2 can inhibit ferroptosis in alcoholic liver disease by regulating SLC7A11 and HO-1, and specific knockout of the Nrf2 gene can reduce the expression of SLC7A11 and inhibit the activity of GPX, thus enhancing the sensitivity of PC12 cells to ferroptosis induced by erastin (Qiang et al., 2020; Dong et al., 2021a). In addition, the key iron storage proteins ferritin light chain and heavy chain and GPX4 are regulated by Nrf2. Therefore, Nrf2 reduces the damage caused by oxidative stress by regulating antioxidant enzymes and iron metabolism. Since ferroptosis depends on the accumulation of lipid peroxides and iron, the inhibition of Nrf2 may significantly increase the sensitivity to ferroptosis. Thus, Nrf2 can significantly affect the development of diabetes.

Recently, some researchers have shown that noncoding RNA myocardial infarction-related transcripts (lncRNA MIAT) related to diabetic microvascular dysfunction can affect the development of diabetes and its complications by regulating the expression of Nrf2. LncRNA MIAT, which is also known as retinal noncoding RNA2 or Gomafu, was first shown to be expressed in mototic progenitor cells and retinal progenitor cells. The expression of lncRNA MIAT is decreased with increasing levels of BUN, which is a biomarker of renal function, and the expression level of lncRNA MIAT is negatively correlated with diabetic renal function and may also be a biomarker of DKD. Decreased expression of the lncRNA MIAT promoted the occurrence of ferroptosis by downregulating the expression of Nrf2 and Nrf2 in the nucleus, reducing cell viability, damaging renal tubular epithelial cells and exacerbating the progression of DKD (Yan et al., 2015; Zhou et al., 2015; Ji et al., 2020; Xiao et al., 2021; Wang Z. et al., 2022).

In DR, lncRNA metastasis-associated lung adenocarcinoma transcript 1 (MALAT1) not only regulates the antioxidant defense against DR through Keap1 and Nrf2 but also promotes transcription by increasing its own binding to the Keap1 promoter (Radhakrishnan and Kowluru, 2021). The increase in Keap1 levels prevents the regulatory factor Nrf2 from moving into the nucleus to initiate the transcription of antioxidant response genes, resulting in the retina being unable to protect itself due to the increase in oxidative stress, which leads to the exacerbation of ferroptosis.

In addition, miR-23c can also be a target of lncRNA MALAT1 in DKD model rats and high glucose-treated HK-2 cells (Zhang et al., 2019a). The overexpression of lncRNA MALAT1 can inhibit the expression of miR-23c, upregulate ELAVL1 expression, and increase the expression of its downstream proteins NLRP3 and Caspase-1 and the proinflammatory cytokine IL-1β, thus regulating the occurrence of ferroptosis and apoptosis caused by Nrf2 and exacerbating the development of DKD, which indicates that lncRNA MALAT1 can affect the development of diabetes by regulating Nrf2, a key regulator of ferroptosis (Gordon et al., 2018; Huang et al., 2021).

**FIGURE 2:**
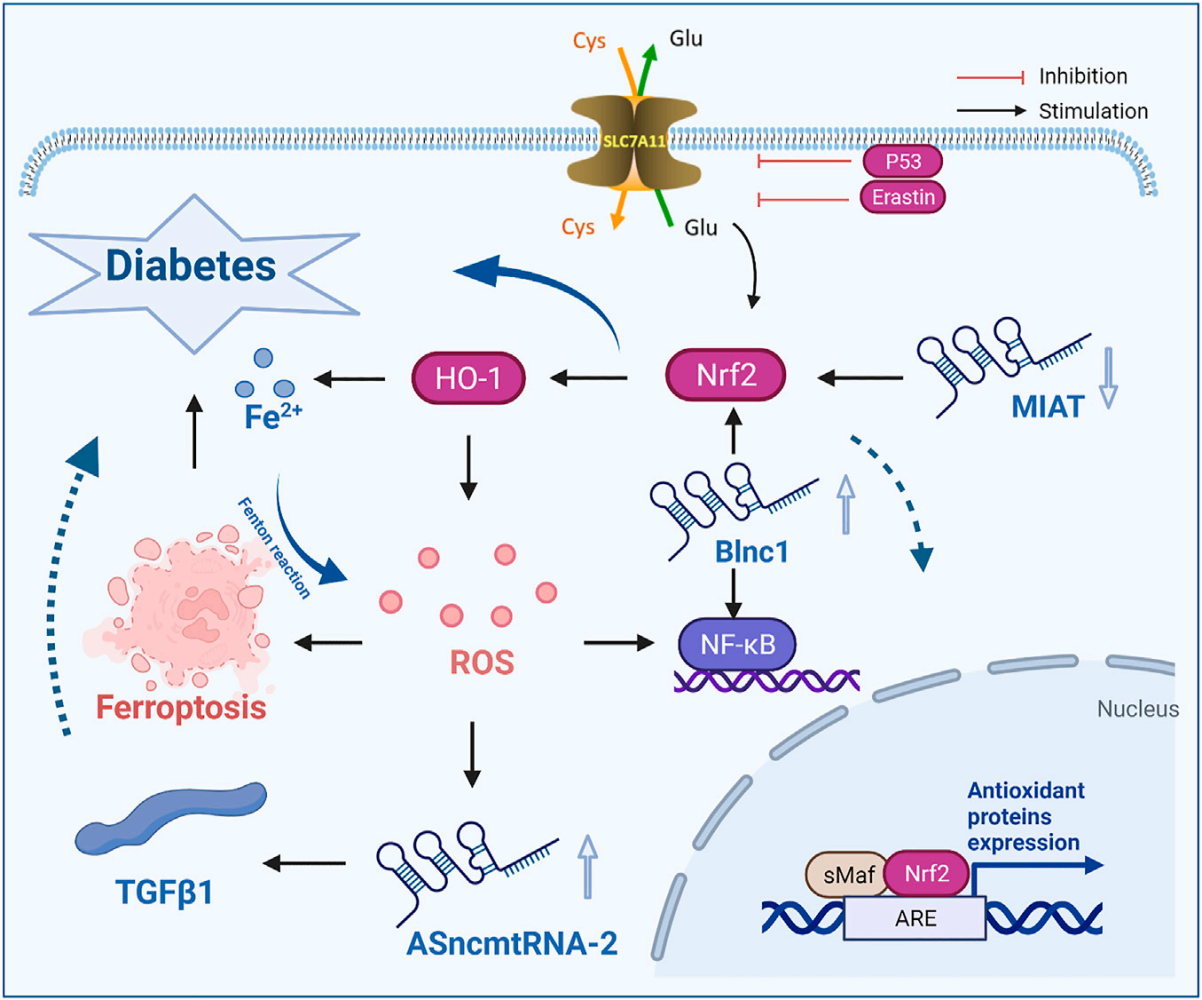
LncRNAs influence the occurrence and development of diabetes by regulating the ferroptosis-inducing factor ROS and the key factor Nrf2. ROS, reactive oxygen species; TGFβ1, transforming growth factor-β1; lncRNA MIRT, myocardial infarction related transcripts; Cys, cystine; Glu, glutamate.

Therefore, lncRNA ASncmtRNA-2, lncRNA NEAT1, lncRNA TUG1 and lncRNA Blnc1 reduce the damage associated with diabetes and its complications by regulating the ferroptotic factor ROS, while lncRNA MIAT and lncRNA MALAT1 inhibit ferroptosis by regulating the key regulatory factor Nrf2, thus slowing the development of diabetes and its complications (Figure 2).

### LncRNAs regulate ferroptosis and affect the development of diabetes and its complications by regulating the NF-κB signaling pathway

Iron-chelating agents affect ferroptosis and diabetes and its complications through the nuclear factor kappa B (NF-κB) signaling pathway, and lncRNAs play a role in the NF-κB signaling pathway of ferroptosis, affecting the occurrence and development of diabetes. It has been shown that deferiprone reduces the high expression of NF-κB and COX2 in cardiomyocytes of DCM rats by interfering with the inflammatory signaling pathway, thus alleviating the development of DCM(L et al., 2019; Li et al., 2020c; Cheng et al., 2021). Furthermore, deferiprone treatment can significantly inhibit the expression of tenascin C and collagen Ⅳ in DCM rats and reduce the level of myocardial fibrosis, which inversely suggests that ferroptosis promotes the development of DCM.

In patients with diabetes, GPX4 can regulate the NF-κB signaling pathway in the context of ferroptosis. GPX4 can reduce the inflammatory response by inhibiting lipoxygenase and reducing the accumulation of ROS(Zou et al., 2017; Anthonymuthu et al., 2021; Yan et al., 2021). In mesangial cells, endothelial cells and renal tubular epithelial cells cultured with high glucose, lncRNAs were involved in the inflammatory response. Gm4419 is a lincRNA located on chromosome 12 (Chr12:21417911-21419803, 1730 bp). Gm4419 is mainly a cytoplasmic lncRNA in MC, which plays a role in gene regulation. Both NF-κB subunits P50 and p65 have a binding relationship with Gm4419. After overexpression of Gm4419, the protein expression levels of P50 and p65 were significantly increased, and the activation of NF-κB was enhanced. After silencing Gm4419, the protein expression levels of P50 and p65 were significantly decreased, the activation of NF-κB was attenuated, and the effect of overexpression of Gm4419 on McP-1 expression was blocked by specific P50 inhibitor. LncRNA Gm4419 may increase the inflammatory response and promote the development of DKD through the NF-κB/NLRP3 inflammatory signaling pathway. Through RNA sequencing and bioinformatics methods, it was found that lincRNA Gm4419 was the only factor that bound to NF-κB among 12 lncRNAs with abnormal DKD expression. It was further confirmed that knocking down Gm4419 in MCs cultured with high glucose could significantly improve inflammation, fibrosis and proliferation (Yi et al., 2017; Ma et al., 2019; Li et al., 2020a). Thus, lncRNA Gm4419 can improve the symptoms of diabetes and its complications by regulating the NF-κB signaling pathway and the ferroptosis pathway.

LncRNA prevention of diabetic neuropathy, oxidative stress and renal fibrosis has attracted more and more attention. The expression of lncRNA Blnc1 in the blood of DN patients and rats is improved, and inactivation of lncRNA Blnc1 can aggravate adipose tissue inflammation and fibrosis, insulin resistance and hepatic steatosis. Studies have confirmed that when lncRNA Blnc1 is upregulated, the levels of Nrf2, HO-1 and NF-κB, which are key proteins, are significantly reduced in HK-2 cells, and this effect can be reversed by lncRNA Blnc1 inhibitors (Zhao et al., 2018). Studies have shown that the lncRNA Blnc1 can reduce the occurrence of ferroptosis through the Nrf2/HO-1 and NF-κB pathways and can be used as a novel regulator of inflammation, oxidative stress and fibrosis in DN.

**FIGURE 3:**
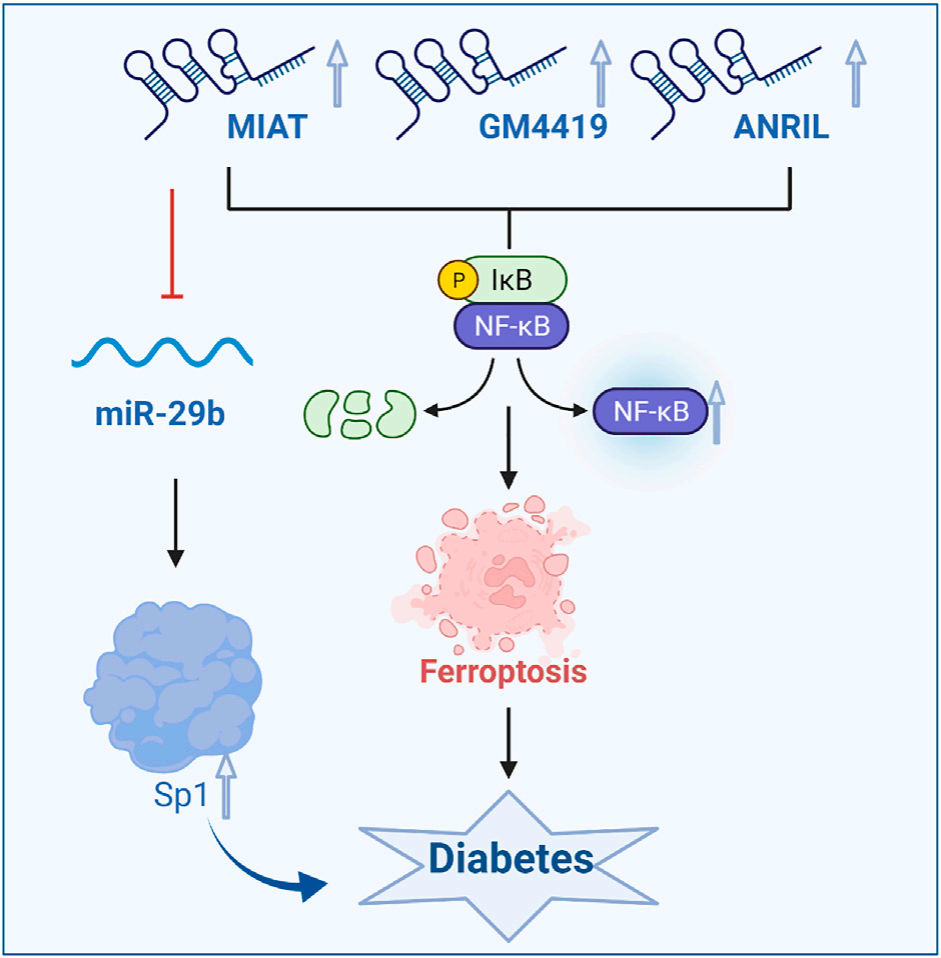
LncRNAs influence the occurrence and development of diabetes by regulating the NF-κB signaling pathway and ferroptosis. GPX4, glutathione peroxidase 4.

A large number of studies have shown that the lncRNA MIAT is mainly expressed in heart and brain tissues, but abnormal expression of this factor affects cell proliferation, apoptosis and migration in many diseases, such as myocardial infarction, microvascular dysfunction, and diabetes. A study showed that increased expression of the lncRNA MIAT in Müller cells in diabetic rats activated the NF-κB signaling pathway, which led to ferroptosis (Lange et al., 2012; Zhou et al., 2017). In addition, the lncRNA MIAT can bind and directly inhibit miR-29b, indirectly promote the expression of the downstream protein Sp1, and then regulate the apoptosis in Müller cells, thus affecting the occurrence of diabetes and its complications. In patients with diabetic retinopathy, under high glucose conditions, lncRNA MIAT can increase the level of activated caspase-3 protein, downregulate the level of phosphorylated protein kinase B, reduce retinal cell apoptosis and alleviate the exacerbation of ferroptosis, thus alleviating the development of diabetes (Zhang et al., 2017; Li et al., 2018). As a competitive endogenous lncRNA, the lncRNA MIAT forms a feedback pathway with vascular endothelial growth factor and miR-150-5p to regulate endothelial cell function. Moreover, the lncRNA MIAT can also bind to NF-κB to mediate ferroptosis, thereby inhibiting endothelial cell proliferation, migration and angiogenesis and exacerbating diabetic retinal microvascular dysfunction. Conversely, silencing the lncRNA MIAT gene can improve diabetic retinal microvascular dysfunction. In addition, there is a regulatory relationship between the lncRNA MIAT and miR-29b. Inhibiting lncRNA MIAT expression can significantly reverse the low expression of miR-29b, high expression of the miR-29b target protein Sp1 and apoptosis induced by high glucose. Studies have shown that lncRNA ANRIL can also enhance the effect of vascular endothelial growth factor on vascular proliferation and slow the degree of ferroptosis by activating the NF-κB signaling pathway, thus alleviating diabetic macroangiopathy (Figure 3)(Cai and Jiang, 2020; Dong et al., 2021b).

Therefore, lncRNAs that regulate the NF-κB signaling pathway affect ferroptosis and the development of diabetes and its complications to a certain extent. In future studies, lncRNA Gm4419, lncRNA Blnc1, and lncRNA MIAT can be used to regulate ferroptosis as a new strategy to treat diabetes and its complications.

### Other lncRNAs affect the development of diabetes and its complications by regulating ferroptosis

In addition to the abovementioned lncRNAs, an increasing number of lncRNAs have been shown to treat diabetes and its complications by inhibiting ferroptosis. For example, lncRNA-ZFAS1 plays an important role in the pathological process of DCM. Inhibiting lncRNA-ZFAS1 decreased collagen deposition, resulting in the sponging of miR-150-5p and the downregulation of cyclin D2 to slow ferroptosis in cardiomyocytes and the progression of DCM(Ni et al., 2021). In addition, lncRNA Meg3 is an important mediator of ischemic stroke. When lncRNA Meg3-p53 was knocked down, the levels of GPX4 were increased by regulating the transcription and expression of GPX4, reducing ROS production, and reducing the damage caused by endoplasmic reticulum stress. Moreover, in response to Meg3-siRNA, the effects of ferritin lysis were reversed, which exacerbated the occurrence of ferroptosis and affected the development of diabetes and its complications. In addition, silencing miR-182-5p and miR-378a-3p can also inhibit ferroptosis and attenuate renal injury induced by ischemia-reperfusion injury in rats (You et al., 2016; Chen et al., 2021b; Li, 2021). LncRNAs such as lncRNA-ZFAS1, lncRNA Meg3, miR-182-5p and miR-378a-3p have been shown to affect the occurrence and development of diabetes and its complications by regulating the ferroptosis pathway. However, additional studies are needed to examine the roles of other lncRNAs in ferroptosis and their targets to influence the occurrence and development of diabetes and its complications, and this is expected to become an effective strategy to alleviate diabetes and its complications and bring new directions for clinical treatments. In addition, the importance of ferroptosis regulated by microRNAs and circular RNAs in diabetic complications should not be ignored. The upregulation of microRNAs such as Mir-150-5p in high glucose treated fibroblasts leads to significantly enhanced levels of the proinflammatory cytokine NF-κB, thereby influencing the development of diabetes through ferroptosis NF-κB signaling pathway. Circular RNAs such as CircANRIL can promote the inflammation and apoptosis of HK2 cells treated with LPS through Mir-9/NF-κB pathway. Therefore, ferroptosis regulated by microRNAs and circular RNAs also plays an important role in diabetic complications (Jin et al., 2020; Jin, 2021).

## Conclusion and prospects

Diabetes and its complications have become a serious threat to human health. Ferroptosis is a kind of cell death that is different from apoptosis, autophagy, and cell death caused by an imbalance in iron metabolism and significantly affects the occurrence and development of diabetes and its complications. LncRNAs can be detected in human body fluids and have good specificity, and these factors can affect the development of diabetes by regulating the ferroptosis pathway. For example, lncRNAs can affect the development of diabetes by regulating the ferroptosis inducer ROS, the key regulatory factor Nrf2 and the NF-κB signaling pathway. Therefore, an in-depth study of the molecular mechanism of lncRNA-regulated ferroptosis in diabetes will shed new light on the prevention and treatment of diabetes.

At present, research on the effect of lncRNAs on diabetes and its complications by regulating ferroptosis is still in the early stage, and there are still many problems to be solved. First, we should investigate whether there are other key lncRNAs that regulate the ferroptosis signaling pathway to affect the development of diabetes and its complications. The second is to explore whether there are lncRNA targets that regulate the upstream inducer of ferroptosis to affect diabetes and its complications. The third is to explore the biological nuances of the role of lncRNAs in diabetes by regulating ferroptosis and the *in vivo* study of cell limiting function loss. Finally, future research can also regulate the occurrence of ferroptosis by focusing on miRNAs and circRNAs, and there is great potential for the treatment of diabetes and its complications.

## Abbreviations

DN, diabetic nephropathy; DCM, diabetic cardiomyopathy; DOP, diabetic osteoporosis; DR, diabetic retinopathy; DPN, diabetic peripheral neuropathy; Fer-1, ferroptosis-1; GSH, glutathione; GPX4, glutathione peroxidase 4; lncRNAs, long noncoding RNAs; MDA, malonaldehyde; ncRNAs, noncoding RNAs; miRNAs, microRNAs; system xc-, cystine/glutamate anti-porter system xc-; PUFA, polyunsaturated fatty acid; ROS, reactive oxygen species; T2DM, type 2 diabetes mellitus; lncRNA ASncmtRNA-2, antisense mitochondrial noncoding RNA-2; L-NAME, NG-nitro-l-arginine methyl ester; TGFβ1, transforming growth factor-β1; HK-2, human kidney-2; Nrf2, nuclear factor erythroid 2-related factor 2; HO-1, Heme oxygenase-1; lncRNA MIAT, noncoding RNA myocardial infarction-related transcripts; MALAT1, metastasis-associated lung adenocarcinoma transcript 1; NF-κB, nuclear factor kappa B.
